# Redox Imbalance in Nasal Epithelial Cells of Primary Ciliary Dyskinesia Patients

**DOI:** 10.3390/antiox13020190

**Published:** 2024-02-02

**Authors:** Ana Reula, Silvia Castillo-Corullón, Miguel Armengot, Guadalupe Herrera, Amparo Escribano, Francisco Dasí

**Affiliations:** 1 Valencia University Clinical Hospital Research Foundation, Instituto de Investigación Sanitaria INCLIVA, Avda. Menéndez y Pelayo, 4, 46010 Valencia, Spain; ana.reula@uchceu.es (A.R.); castillo_sil@gva.es (S.C.-C.); amparo.escribano@uv.es (A.E.); 2 Rare Respiratory Diseases Research Group, Department of Physiology, School of Medicine, University of Valencia, Avda. Blasco Ibáñez, 17, 46010 Valencia, Spain; 3 Biomedical Sciences Department, CEU-Cardenal Herrera University, 12006 Castellón, Spain; 4 Molecular, Cellular, and Genomic Biomedicine Group, IIS La Fe, 46026 Valencia, Spain; miguel.armengot@uv.es; 5 Paediatrics Unit, Department of Pediatrics, Obstetrics and Gynecology, Hospital Clínico Universitario Valencia, University of Valencia, 46022 Valencia, Spain; 6 ENT Unit, Department of Surgery, School of Medicine, Hospital La Fe, University of Valencia, Avda. Blasco Ibáñez, 17, 46010 Valencia, Spain; 7 Flow Cytometry Unit, Fundación Investigación Hospital Clínico Valencia, Instituto de Investigación Sanitaria INCLIVA, University of Valencia, Avda. Menéndez y Pelayo, 4, 46010 Valencia, Spain; guadalupe.herrera@uv.es

**Keywords:** primary ciliary dyskinesia, rare respiratory diseases, oxidative stress, nitric oxide, hydrogen peroxide

## Abstract

**Background:** Primary Ciliary Dyskinesia (PCD) represents a rare condition marked by an abnormal mobility pattern of cilia and flagella, resulting in impaired mucociliary clearance. This deficiency leads to recurrent infections and persistent inflammation of the airways. While previous studies have indicated heightened oxidative stress levels in the exhaled breath condensate of pediatric PCD patients, the assessment of oxidative stress within the affected respiratory tissue remains unexplored. **Aims:** To assess the oxidative status of human nasal epithelial cells (NECs) in PCD patients. **Methods:** Thirty-five PCD patients and thirty-five healthy control subjects were prospectively included in the study. Levels of reactive oxygen species (ROS), reactive nitrogen species (RNS), glutathione (GSH), intracellular Ca^2+^, plasma membrane potential, and oxidative damage in lipids and proteins were measured. In addition, apoptosis and mitochondrial function were analyzed by flow cytometry in NECs. **Results:** NECs from PCD patients showed reduced levels of apoptosis (*p* = 0.004), superoxide anion (O_2_^−^, *p* = 0.018), peroxynitrite (ONOO^−^, *p* = 0.007), nitric oxide (NO, *p* = 0.007), mitochondrial hydrogen peroxide (mtH_2_O_2_, *p* < 0.0001), and mitochondrial superoxide anion (mtO_2_^−^, *p* = 0.0004) and increased mitochondrial mass (*p* = 0.009) compared to those from healthy individuals. No significant differences were observed in oxidized proteins (*p* = 0.137) and the oxidized/reduced lipid ratio (*p* = 0.7973). The oxidative profile of NEC cells in PCD patients, according to their ciliary motility, recurrent otitis, recurrent pneumonia, atelectasis, bronchiectasis, and situs inversus, showed no statistically significant differences in the parameters studied. Conversely, patients with chronic rhinosinusitis exhibited lower levels of ONOO^−^ than PCD patients without this condition, with no significant differences related to other symptoms. **Conclusions:** Our findings strongly suggest the presence of a redox imbalance, specifically leaning toward a reductive state, in PCD patients.

## 1. Introduction

Primary Ciliary Dyskinesia (PCD) is a rare autosomal recessive disorder (ORPHA:244) with an incidence of 1 in 7500 [1]. This condition is characterized by impaired cilia motility, resulting in inefficient mucociliary transport. The consequence is the accumulation of secretions in the upper and lower respiratory tract, making individuals with PCD prone to recurrent infections in the ears, nose, and lungs. Additionally, the disorder predisposes patients to chronic neutrophilic inflammation in the lower respiratory tract. The compromised ciliary function in PCD significantly impacts the normal clearance of mucus, contributing to the clinical manifestations associated with the disease [2].

In PCD lungs, activated neutrophils, which are notably abundant, play a crucial role in the inflammatory response. These neutrophils release multiple proteases and generate substantial quantities of reactive oxygen and nitrogen species (ROS and RNS, respectively). The increased levels of ROS and RNS contribute to an environment that has the potential to eliminate agents causing inflammation [3].

Oxidative stress arises from an imbalance in the body’s pro-oxidant and antioxidant mechanisms toward the former. The accumulation of ROS and RNS characterizes this condition. These molecules have the capacity to interact with nucleic acids, lipids, and proteins, inducing their oxidation and resulting in irreversible damage to cells. The consequential effect is the initiation of tissue destruction, underscoring the profound impact of oxidative stress on cellular components and overall tissue integrity [4].

A correlation between oxidative stress and chronic inflammation has been established, highlighting the intricate interplay between these two physiological processes. Previous studies have demonstrated that oxidative stress triggers the activation of transcription factors, leading to the altered expression of genes associated with inflammatory pathways [4]. Substantial evidence supports the notion that oxidative stress is implicated in chronic inflammatory conditions affecting the respiratory system, including Chronic Obstructive Pulmonary Disease (COPD) [5], Cystic Fibrosis (CF) [6], and Alpha-1 Antitrypsin Deficiency (AATD) [7,8,9], underscoring the significance of oxidative stress as a contributing factor to the pathogenesis of various respiratory inflammatory disorders.

In previous investigations, elevated levels of 8-isoprostane, an established marker of oxidative stress, have been observed in the exhaled air condensate of children affected by Primary Ciliary Dyskinesia (PCD) [10,11]. However, the assessment of oxidative stress within the affected tissue remains unexplored, prompting the need to examine the oxidative stress profile within the cells comprising the ciliated respiratory epithelium, which is particularly relevant in understanding its role in the characteristic chronic inflammatory processes associated with PCD. Given the compromised mucociliary clearance in PCD patients, which predisposes them to persistent inflammation, the connection between chronic inflammation and oxidative stress becomes a focal point of interest [2]. The hypothesis postulated in this study is that oxidative stress is involved in the pathophysiology of PCD.

Consequently, this study aims to unravel the oxidative stress profile in human nasal epithelial cells (NECs) from PCD patients and healthy control individuals. By focusing on the specific cells affected by this disorder, we aim to contribute to a more comprehensive understanding of the oxidative dynamics intrinsic to PCD pathology, potentially shedding light on novel avenues for therapeutic intervention in this challenging and rare respiratory disease.

## 2. Materials and Methods

### 2.1. Patients

A total of 35 PCD patients (17 males/18 females) and 35 healthy control volunteers (20 males/15 females) admitted to the Paediatrics Unit of the Hospital Clínico Universitario Valencia (HCUV) and the Otorhinolaryngology Unit of the Hospital General Universitario Valencia (HGUV) were prospectively included in the study.

Inclusion criteria were the following: (i) patients diagnosed with PCD according to the European Respiratory Society guidelines [12,13] showing signs and symptoms characteristics of the disease (chronic productive cough, chronic rhinorrhea) with or without neonatal distress, *situs inversus,* or bronchiectasis with confirmed diagnosis by ciliary ultrastructure determined by electronic microscopy [14] and ciliary beat pattern measured by high-speed video microscopy analysis [15] and (ii) control individuals with no history or clinical findings that suggested a respiratory pathology, allergies, or rhinosinusitis. Exclusion criteria were (i) smoking; (ii) patients with conditions associated with oxidative stress, such as COPD, rheumatoid arthritis, lupus erythematosus, inflammatory bowel disease, diabetes mellitus, cardiac dysfunction, and neoplasms; (iii) active fever or infection; (iv) surgery less than three months before the sample collection; (v) intense physical exercise less than two days before sample collection; (vi) obesity; and (vii) treatment with antioxidants three months before sample collection.

The study received approval from the research ethics committees of HCUV and HGUV. Before participation, detailed procedures were communicated to all participants, and written informed consent was obtained before being included in the study.

### 2.2. Anamnesis and Physical Examinations

Demographic (age and sex) and clinicopathological data were collected from each individual participating in the study in an interview carried out at their inclusion (Table 1, Table 2 and Table 3). Anthropometric measurements were performed using standard techniques. Body mass index was calculated as kg.m^−2^. In addition, before collecting the biological sample, a clinical examination was carried out to evaluate the current state of health of the participants. All PCD patients included in the study were clinically stable according to their physical status and free of respiratory exacerbations for at least three months before being included in the study.

### 2.3. Sample Collection

NECs from PCD patients and control individuals were obtained by nasal brushing as previously described [16]. A cytology brush (Covaca SA CE2005, Madrid, Spain) was inserted into the patient’s nostril, gently rubbing the middle nasal cornet (the area with the highest density of ciliated cells). The collected samples were transported from the participating hospitals to the laboratory at the Faculty of Medicine in a container at 4 °C in 10 mL of Medium 199 (1X) with salts of Hanks, L-Glutamine, HEPES 25 mM (Gibco; Madrid, Spain (Invitrogen 22350-029)), supplemented 1% with Penicillin/Streptomycin (Gibco (Invitrogen; Madrid, Spain 15070-063)). Samples were filtered with a CellTrics^®^ 50 μm filter (25004-0042-2317 Sysmex; Barcelona, Spain) and counted (FACSVerse flow cytometer; BD Biosciences; Madrid, Spain) before any flow cytometry determinations.

### 2.4. Apoptosis, Oxidative/Nitrosative Stress, Mitochondrial Assessment, and Damage to Biomolecules

Apoptosis, oxidative/nitrosative stress, mitochondrial assessment, and damage to biomolecule parameters were measured by flow cytometry as previously described by our research group [16]. A laboratory bench step-by-step protocol used to measure these parameters can be found at the following link: https://www.mdpi.com/2077-0383/10/6/1172/s1 (accessed on 30 January 2024).

#### 2.4.1. Cytometric Measurements

Cytometric assays were carried out in a FACS Verse flow cytometer (BD Biosciences, San Jose, CA, USA), except the lipid oxidized/lipid reduced ratio, which was carried out using the FacsAria III cytometer (BD Biosciences), and kinetic analyses of NO, ONOO, and Ca^2+^ generation, which were carried out using the LSR Fortessa X-20 (BD Biosciences). Results are expressed as mean fluorescence intensity in arbitrary units. NECs were gated from other contaminating cells according to their morphology (measured by the forward scatter) and internal complexity (measured by the side scatter). Afterward, a gate was applied to identify specific populations of individual cells.

The fluorescent probes Bis-(Dibutylbarbituric Acid) Trimethine Oxonol (DIBAC), FLUO-4, 5-chloromethyl fluorescein diacetate (CMFDA), 4-Amino-5-Methylamino-2′7′-Difluorescein Diacetate (DAF-FM DA), 2′7′- dichlorodihydrofluorescein (DCFH), MitoSOXTM Red (MitoSOX), Mitotracker Green, BODIPY 665/676 C 11 (B665), Tetramethylrhodamine (TMRM), and Dihydrorhodamine 1,2,3 (DHR1,2,3) were from Molecular Probes (Eugene, OR). Dihydroethidium (HE), Propidium Iodide (PI), fluorescein 5-thiosemicarbazide (FTC), 4′, 6′-Diamidino-2- phenylindole dihydrochloride (DAPI), and mitochondria peroxy yellow 1 (MitoPY 1) were from Sigma (San Luis, MO, USA). Annexin V was purchased from Inmunostep (Salamanca, Spain). The inductors tert-Butyl hydroperoxide (t-BHP), Ionomycin ionophore, Diethyl maleate (DEM), Plumbagin (PB), Carbonyl cyanide 4- (trifluoromethoxy)phenylhydrazone (FCCP), and Menadione were from Sigma (San Luis, MO, USA). NOR-1 was from Santa Cruz Biotechnology (Dallas, TX, USA).

#### 2.4.2. Determination of Cellular Viability

Cellular viability was determined by adding a DNA-binding dye 4′,6-diamidino-2-phenylindole (DAPI) into tubes with dihydroethidium (HE), 2′-7′dichlorofluorescin diacetate (DCFH), Dihydrorhodamine 123 (DHR1,2,3), Diaminofluorescein-FM diacetate (DAF-FM/DA), 5-chloromethyl fluorescein diacetate (CMFDA), Tetramethylrhodamine, methyl ester (TMRM), MitoTracker Green, MitoSOX, MitoPY 1, FLUO-4, BODIPY 665 (B665), and FTC. In contrast, propidium iodide (PI) was used with Bis (1,3-dibutyl barbituric acid) trimethine oxonol (DIBAC). Dead cells were identified and excluded from further analysis.

#### 2.4.3. Reactive Oxygen Species and Reactive Nitrogen Species Assessment

Superoxide anion (O_2_^−^) measurement was based on HE oxidation. HE was added to the NECs at a final concentration of 2.5 ug/mL and incubated for 20 min at 37 °C, and the fluorescence generated was measured by flow cytometry. Once the basal production of O_2_^−^ had been achieved, a 2.2 ug/mL dose of plumbagin (PB), a superoxide generator, was added to the sample in order to induce maximal production of O_2_. Fluorescence using a FACS Verse Cytometer (excitation laser 488 nm; detector: 700/54665 LP).

Peroxynitrite (ONOO^−^) production was assessed using DHR1,2,3. DHR was added to a final concentration of 100 µM and incubated for 20 min at 37 °C, and the generated fluorescence was measured by flow cytometry. Once the basal production of ONOO^−^ was achieved, NOR-1 (final concentration: 16 µg/mL) was added to the cells to induce maximal production of ONOO^−^. Fluorescence was measured using an LSR Fortessa X-20 Cytometer (excitation laser 488 nm; detector: 530/30505 LP).

Nitric oxide (NO) production was assessed using the fluorescent probe DAF-FM/DA). DAF was added at a final concentration of 1 µM and incubated for 20 min at 37 °C, and the generated fluorescence was measured by flow cytometry. Once the basal production of NO was achieved, NOR-1 (final concentration: 16 µg/mL) was added to the cells to induce maximal production of NO. Fluorescence was measured using an LSR Fortessa X-20 Cytometer (excitation laser 488 nm; detector: 530/30505 LP).

The assay to detect intracellular peroxides was based on DCFH oxidation. DCFH was added at a final concentration of 2.5 µg/mL and incubated for 20 min at 37 °C, and the fluorescence generated was measured by flow cytometry. Once basal production of intracellular peroxides was achieved, a 100 µM dose of t-butyl-hydroperoxide (t-BHP) was added to the sample to induce maximal production of intracellular peroxides. Fluorescence was measured using a FACS Verse Cytometer (excitation laser 488 nm; detector: 527/32507 LP).

#### 2.4.4. Reduced Glutathione Assessment

Reduced glutathione (GSH) assessment was based on CMFDA oxidation. CMFDA was added to a final concentration of 25 nM and incubated for 20 min at 37 °C, and the generated fluorescence was measured by flow cytometry. Once basal production of intracellular peroxides was achieved, a 20 mM dose of diethyl maleate (DEM) was added to the sample to induce maximal production of GSH. Fluorescence was measured using a FACS Verse Cytometer (excitation laser 488 nm; detector: 527/32507 LP).

#### 2.4.5. Plasmatic Membrane Potential Assessment

Plasma membrane potential was evaluated using the fluorescent probe Bis-(Dibutylbarbituric Acid) Trimethine Oxonol (DIBAC). NECs were incubated with DIBAC (final concentration: 1.2 μM) for 20 min at 37 °C, and fluorescence was measured by flow cytometry with the appropriate settings. Fluorescence was measured using a FACS Verse Cytometer (excitation laser 488 nm; detector: 527/32507 LP).

#### 2.4.6. Mitochondrial Assessment

Mitochondrial hydrogen peroxide (mtH_2_O_2_) was assessed using MitoPY. NECs were incubated with MitoPY (final concentration: 4 μM) for 20 min at 37 °C, and the fluorescence generated was measured by flow cytometry. Once basal production of mtH_2_O_2_ was achieved, a 100 µM dose of t-BHP was added to the sample to induce maximal production of mtH_2_O_2_. Fluorescence was measured using a FACS Verse Cytometer (excitation laser 488 nm; detector: 527/32507 LP).

Mitochondrial superoxide anion (mtO_2_^−^) was assessed using MitoSOX. NECs were incubated with MitoSOX (final concentration: 640 nM) for 20 min at 37 °C, and the fluorescence generated was measured by flow cytometry. Once basal production of mtO_2_^−^ was achieved, a 2.2 µg/mL dose of PB was added to the sample to induce maximal production of mtO_2_^−^. Fluorescence was measured using a FACS Verse Cytometer (excitation laser 488 nm; detector: 700/54665 LP).

Mitochondrial mass was assessed using Mitotracker Green. NECs were incubated with Mitotracker Green (final concentration: 78 nM) for 20 min at 37 °C, and the fluorescence generated was measured by flow cytometry. Fluorescence was measured using a FACS Verse Cytometer (excitation laser 488 nm; detector: 527/32507 LP).

Mitochondrial membrane potential was determined using TMRM. NECs were incubated with TMRM (final concentration: 640 nM) for 20 min at 37 °C, and the fluorescence generated was measured by flow cytometry. Once basal production of mtO_2_^−^ was achieved, a 52 µM dose of carbonyl cyanide 4-(trifluoromethoxy)phenylhydrazone (FCCP), an uncoupling agent of the mitochondrial electron transport chain, was added to the sample as a positive control. Fluorescence was measured using a FACS Verse Cytometer (excitation laser 488 nm; detector: 586/42560 LP).

#### 2.4.7. Intracellular Calcium Assessment

Intracellular calcium (iCa^2+^) assessment was performed using FLUO-4. NECs were incubated with FLUO-4 (final concentration: 0.5 µM) for 20 min at 37 °C, and the fluorescence generated was measured by flow cytometry. Once basal production of mtO_2_^−^ was achieved, a 50 µM dose of ionomycin was added to the sample as a positive control. Fluorescence was measured using an LSR Fortessa X-20 Cytometer (excitation laser 488 nm; detector: 530/30505 LP).

#### 2.4.8. Markers of Oxidative Damage to Biomolecules

Lipid peroxidation was assessed using the lipophilic probe BODIPY 665/676 dye (B665). NECs were incubated with B665 (final concentration: 800 nM) for 30 min at 37 °C, and fluorescence was measured by flow cytometry with the appropriate settings. A positive control using t-BHP (100 µM) was also included in the assay. Fluorescence was measured using a FACS Aria III Cytometer (excitation laser 488 nm and 635 nm; detector: 588/42556 LP and 780/60735 LP).

Protein oxidation (carbonylation) levels were measured using FTC. NECs were incubated with FTC (final concentration: 800 nM) for 20 min at 37 °C, and fluorescence was measured by flow cytometry with the appropriate settings. A positive control using Menadione (1 mM) was also included in the assay. Fluorescence was measured using a FACS Verse Cytometer (excitation laser 488 nm; detector: 527/32507 LP).

#### 2.4.9. Apoptosis Assay

Apoptosis status was determined using Annexin V. NECs were incubated in the dark for 15 min at room temperature with Annexin V, PI, and Annexin V-binding buffer (previously diluted to 1/10 in PBS). After incubation, 300 μL of the 1/10 Annexin V-binding buffer was added to the dilution. Fluorescence was measured using a FACS Verse Cytometer (excitation laser 488 nm; detector: 527/32507 LP).

### 2.5. Statistical Analysis

Demographic data were expressed as mean and standard deviation (SD). To identify group differences, categorical variables were analyzed using contingency tables and the chi-square (χ2) test. Since both the number of patients and controls was higher than 30, normality was assumed. Otherwise, the Shapiro–Wilk test for small samples was used. If data followed the normal distribution, the means between groups were compared using the *t*-test; otherwise, the Mann–Whitney test was used. The Welch ANOVA (normality) or the Kruskal–Wallis (no normality) tests were used for multiple comparisons of means.

A two-tailed *p*-value lower than 0.05 was considered statistically significant. Statistical analyses were performed using GraphPad Prism 9.4.0 Software (GraphPad, La Jolla, CA, USA) and R for Windows (Boston, MA, USA).

## 3. Results

### 3.1. Demographic and Clinical Data

Demographic and clinical–pathological characteristics of PCD patients and controls included in the study are shown in Table 1, Table 2 and Table 3. No significant differences were observed in age (*p* = 0.226), sex (*p* = 0.632), and body mass index (*p* = 0.520) between groups. In our sample, 9 patients (26%) presented situs inversus, 31 patients (89%) showed chronic cough, 15 patients (43%) presented bronchiectasis, 11 patients had (31%) atelectasis, 10 patients (29%) had asthma, 21 patients suffered from (60%) recurrent otitis, 18 patients presented (51%) chronic rhinosinusitis, and 20 patients have had (57%) pneumonia (Table 1). As mentioned above, all patients were clinically stable according to their physical status and free of respiratory exacerbations for at least three months to avoid confounding factors (such as infections and antibiotic treatment).

In the patients’ group, data regarding ciliary ultrastructure (22 patients) (Table 2) and beating pattern (35 patients) (Table 3) were collected. Six patients (27%) showed a complete loss in both external and internal dynein arms; seven patients (32%) had a complete loss of the inner dynein arms; two patients (9%) presented microtubular disorganization; one patient (5%) had transposition; three patients (14%) had partial dynein deficiency, and three patients (14%) showed normal ciliary ultrastructure (Table 2). Regarding the ciliary beating pattern, nine patients (26%) presented immobile cilia, 11 patients (31%) presented vibratory movement, nine patients (26%) showed reduced amplitude and lack of coordination, and six patients (17%) showed a normal beating pattern although with a reduced frequency (Table 3).

### 3.2. Comparison of the Oxidative Profile of the Nasal Ciliated Epithelium Cells between PCD Patients and Control Individuals

Markers of apoptosis, oxidative stress, the antioxidant system, and mitochondrial oxidative parameters are shown in Table 4.

#### 3.2.1. Apoptosis, ROS/RNS, and Antioxidant Markers

Significantly decreased apoptosis levels were observed in NEC from PCD patients compared to control individuals (*p* = 0.004). PCD patients showed significantly decreased superoxide anion (O_2_^−^; *p* = 0.018), peroxynitrite (ONOO^−^; *p* = 0.0071), and nitric oxide (NO; *p* = 0.0076) levels than the control group. No significant differences were observed between groups in reduced glutathione (GSH) (*p* = 0.867), intracellular calcium (iCa^2+^) (*p* = 0.680), plasma membrane potential levels (*p* = 0.957), and intracellular peroxides (*p* = 0.206).

#### 3.2.2. Mitochondrial Oxidative Parameters

In comparison to the control group, PCD patients showed a significant reduction in mitochondrial H_2_O_2_ (*p* < 0.0001), mitochondrial O_2_^−^ (*p* = 0.0004), and mitochondrial mass (*p* = 0.009). However, there were no significant differences between the groups regarding mitochondrial plasma membrane levels (*p* = 0.200).

#### 3.2.3. Markers of Oxidative Damage

Although oxidized protein levels were higher than control individuals, the differences did not reach statistical significance (*p* = 0.137). Additionally, there were no significant differences between the groups in the lipid oxidized/reduced ratio (*p* = 0.797).

### 3.3. Analysis of the Association between the Oxidative Stress Profile and the Clinicopathological Characteristics of Patients with PCD

After the initial comparative analysis between the groups of patients and controls, a more detailed analysis between the oxidative stress parameters showing statistically significant differences and the patients’ clinicopathological characteristics was carried out. The oxidative profile of ciliated NECs in patients with PCD, according to their ciliary motility, recurrent otitis, recurrent pneumonia, atelectasis, bronchiectasis, and situs inversus, showed no statistically significant differences in any of the parameters studied were observed (Supplementary Tables S1–S6). A significant decrease in ONOO^−^ levels (*p* = 0.013) was observed in PCD patients showing chronic rhinosinusitis, whereas no significant differences were observed in the rest of the analyzed parameters (Supplementary Table S7).

## 4. Discussion

Longitudinal studies indicate that the pulmonary function of PCD patients can remain stable over an extended period when suitable treatments are administered. However, despite this stability, there is an observed decline in lung function with age among PCD patients. Many adults grappling with PCD exhibit a significant burden of disease-related symptoms, ultimately diminishing their overall quality of life [17]. Further research is therefore needed to deepen knowledge of the pathophysiology of the disease.

PCD is a chronic airway disease characterized by impaired mucociliary clearance mechanisms leading to chronic inflammation. Over the years, extensive research has consistently demonstrated a correlation between chronic inflammation and oxidative stress, creating a highly cellular oxidant state that can damage tissues directly by oxidizing the cellular biomolecules or altering critical signaling pathways [4]. Therefore, knowing the underlying mechanisms by which oxidative stress directs pathogenesis is essential to developing more effective therapies [18]. Since the airway’s inflammatory state is one of the primary clinical manifestations of PCD, it is essential to understand the physiological characteristics of the affected tissue, particularly ciliated cells that are part of the nasal respiratory epithelium. Thus, this study aimed to characterize the oxidative profile of nasal epithelial ciliary cells in healthy controls and PCD patients. As far as we know, this is the first study that analyses oxidative/nitrosative stress in NECs from PCD patients.

No significant differences were observed in age (*p* = 0.226), sex (*p* = 0.632), and body mass index (*p* = 0.520) (factors that can modify oxidative stress parameters) between the control and PCD groups (Table 1). The role of age in PCD is an important factor that may significantly influence the clinical manifestation, diagnosis, and management of the disease. Understanding the age-related aspects of PCD is important for several reasons. Firstly, the clinical presentation varies with age, with recurrent infections often observed in childhood, whereas fertility problems are more prominent in the adult age. In our study, none of the patients presented fertility problems, so it was not possible to explore this aspect. Secondly, this age-dependent variability is an important challenge in PCD diagnosis, as symptoms may evolve over time. This aspect was not a problem in our study since, beyond the respiratory clinical manifestations observed, the clinical diagnosis was confirmed by measuring the ciliary beat pattern and ultrastructure.

Our results show a statistically significant decrease in total and mitochondrial O_2_^−^ (*p* = 0.018 and *p* = 0.0004, respectively), mitochondrial H_2_O_2_ (*p* < 0.0001), NO (*p* = 0.007), ONOO^−^ (*p* = 0.007), and in the percentage of apoptotic cells (*p* = 0.004) in NECs from PCD patients. A statistically significant increase in mitochondrial mass (*p* = 0.009) was observed in patients compared to controls. No significant differences were observed in mitochondrial membrane potential (*p* = 0.002), intracellular Ca^2+^ levels (*p* = 0.680), GSH (*p* = 0.867), and plasma membrane potential (*p* = 0.957) (Table 4).

Interestingly, NEC from PCD patients show statistically significantly lower NO levels than controls (*p* = 0.0076). NO is an intra- and intercellular signaling molecule involved in various physiological and pathophysiological processes. Several studies have shown that nNO levels are very low in PCD patients; in fact, they are used as a screening method for diagnosing the disease. Our results agree with those previously reported by other groups that have consistently shown low NO levels in NECs from PCD patients. In these cells, NO regulates ciliary beating frequency via cAMP and cGMP. Thus, cAMP and cGMP activate protein kinases A and G, which phosphorylate dynein light chains, increasing ciliary beating, which is deficient in PCD patients [19]. Our results agree with these previous reports and support the hypothesis that the low nNO levels observed in PCD patients are due to a decreased biosynthesis of the molecule since, at the cellular level, we have observed that these levels are significantly lower.

In NECs derived from PCD patients, a noteworthy reduction in ONOO^−^ levels (*p* = 0.007) and a decrease in apoptosis (*p* = 0.004) is evident compared to the control group. The generation of ONOO^−^ is contingent upon the availability of NO and superoxide O_2_^−^. It has been documented that NO can induce apoptosis in the presence of O_2_^−^ through the peroxynitrite production pathway. In the context of PCD, where levels of NO and O_2_^−^ in NECs are diminished, there is a consequential reduction in ONOO^−^ production, leading to a parallel decrease in apoptosis levels. Peroxynitrite levels have not been previously determined in PCD patients. However, significantly lower ONOO^−^ lowers have been observed in other respiratory pathologies such as Chronic Obstructive Pulmonary Disease [20,21] and bronchial asthma [22]. Altogether, these results shed light on the intricate interplay between reactive oxygen and nitrogen species in the cellular milieu of PCD patients and highlight the importance of NO and peroxynitrite as major effectors in respiratory diseases and airflow obstruction.

In examining mitochondrial dynamics, our findings unveil a paradoxical scenario in PCD patients, characterized by an elevated mitochondrial mass (*p* = 0.009) alongside significantly diminished mtH_2_O_2_ and O_2_^−^ compared to healthy controls (*p* < 0.0001 and *p* = 0.0004, respectively). Within the mitochondria, a specialized enzyme, mitochondria-specific nitric oxide synthase (NOS), generates NO, initiating the production of mtH_2_O_2_ through the autooxidation of ubiquinol. Under normal physiological conditions, mtH_2_O_2_ is involved in signaling cascades. Simultaneously, mitochondrial NO combines with mtO_2_^−^ to form ONOO^−^. Our observations prompt speculation that mitochondrial NOS might act as a peroxynitrite synthase, potentially promoting ONOO^−^ production within the organelle. This conjecture offers a plausible explanation for the observed reduction in mtO_2_^−^ and H_2_O_2_ levels in nasal epithelial cells of PCD patients, adding a layer of complexity to the understanding of mitochondrial dysfunction in the context of this respiratory disorder. Mitochondria have long been recognized for their role in energy production within cells. However, beyond this classical role of mitochondria, recent studies reveal a novel connection between mitochondrial function and the formation and function of cilia. Recent research has demonstrated a significant reduction in mitochondrial DNA (mtDNA) in a specific group of heterotaxy patients. Later investigations extended to manipulating mitochondrial function in diverse organisms, including zebrafish embryos, human fibroblasts, and *Tetrahymena thermophila*, producing heterotaxy-like phenotypes. Furthermore, alterations in cilia length were identified, exhibiting an inverse correlation with changes in mitochondrial function, indicating that as mitochondrial function diminished or increased, cilia length displayed corresponding aberrations [23]. Recently, it has been reported that mitochondrial dysfunction compromises ciliary homeostasis in astrocytes [24].

Compared to control individuals, our investigation did not reveal any significant differences in oxidized proteins (*p* = 0.137) or the ratio of oxidized to reduced lipids (*p* = 0.797) among PCD patients. These outcomes align with the overall conclusion that PCD patients do not exhibit higher levels of oxidative stress compared to controls. However, our findings diverge from those reported by Zihlif et al., who documented increased 8-isoprostane levels in the exhaled breath condensate of PCD children [10]. We attribute these disparities to variations in the measurement of oxidized lipid levels within different compartments. Exhaled breath condensate assessments offer a broad overview of the airway, making it challenging to pinpoint the specific contributions of individual cell types. In contrast, our determinations are conducted directly in ciliated cells, providing a more accurate representation of the oxidative milieu within these cells.

Interestingly, characteristic signs and symptoms associated with PCD did not impact the determined oxidative parameters. No statistically significant differences were noted across the analyzed cases, except for a notable decrease in ONOO^−^ levels in patients with chronic rhinosinusitis (*p* = 0.0129) (Supplementary Tables S1–S7). The clinical significance of these observations is uncertain, as no previous studies have been performed to investigate the influence of these symptoms on the oxidative status of NECs. These findings underscore the complexity of oxidative processes in PCD and warrant further exploration to elucidate the potential interplay between symptoms and oxidative parameters in this clinical context.

In summary, our findings strongly suggest the presence of a redox imbalance, specifically leaning toward a reductive state, in PCD patients. The observed deviation from normal redox homeostasis raises intriguing possibilities about its potential involvement in the pathophysiology of the disease. As indicated by our results, the shift toward a reductive environment in PCD patients emphasizes the significance of investigating the role of reductive stress in understanding the cellular dynamics and molecular mechanisms underlying PCD. Redox imbalance plays an important role in the pathogenesis, progression, and exacerbation of several pulmonary diseases, such as asthma, COPD, and interstitial lung diseases. In these diseases, redox imbalance is associated with the perpetuation of inflammation and the decline of lung function. Understanding the redox mechanisms in these diseases will allow for developing personalized therapeutic strategies since antioxidant strategies to restore redox homeostasis have shown promising results in mitigating oxidative stress-related damage and alleviating symptoms in various respiratory diseases. Our results show that, at the mitochondrial level, PCD patients are characterized by low levels of mtH_2_O_2_, low levels of mtO_2_^−^, and increased mitochondrial mass. At the cellular (cytoplasmatic) level, patients show low cytoplasmatic levels of O_2_^−^, ONOO^−^, and NO. Overall, our results show lower levels of ROS production in NECs from patients compared to controls and an increased mitochondrial mass. Lower levels of ROS in patients’ NECs may indicate a unique REDOX state. Although respiratory diseases are traditionally associated with oxidative stress, our results suggest a different REDOX equilibrium in PCD. Understanding this atypical REDOX environment could provide new insights into the mechanisms underlying respiratory diseases. The increased mitochondrial mass suggests a possible compensatory response in PCD patients. As mentioned above, mitochondria play an important role in cellular energy production and are involved in the regulation of ROS. The altered mitochondrial mass may reflect an adaptive mechanism to maintain cellular function despite lower ROS levels.

Altogether, these results suggest a complex redox imbalance. Treating such a complex scenario would require a comprehensive approach to restore cellular homeostasis. The identification of lower levels of ROS in patients’ cells could encourage the investigation of therapies aimed at modulating ROS. Although antioxidant treatments may not be of immediate application in view of these results, consideration could be given to exploring interventions aimed at redox balance or improving mitochondrial function. However, antioxidant therapies that specifically target the mitochondria, such as MitoQ or SK1, which can replenish the reduced levels of mtH_2_O_2_ and mtO_2_^−^, could be beneficial. Other compounds, such as coenzyme Q10, involved in the mitochondrial electron transport chain may help regulate the mitochondrial function. Mitochondrial biogenesis regulation could be another therapeutic option. Pharmaceutical agents such as PGC-1 alpha activators could help control the increased mitochondrial mass observed. Other strategies, such as supplementation with NO precursors (i.e., L-arginine), may help increase cytoplasmic levels of NO. In addition, implementing anti-inflammatory measures may help reduce the redox imbalance. While antioxidants are typically associated with reducing oxidative stress, certain antioxidants, such as N-acetylcysteine or Vitamin C, also help regulate reductive stress.

In addition, the observed differences in ROS production and mitochondrial mass may serve as potential diagnostic markers for PCD. These markers could be useful in distinguishing PCD patients from healthy individuals, aiding in early detection and personalized treatment strategies.

Finally, we would like to acknowledge three limitations in our study. Firstly, the study’s low sample size is attributed to the rare prevalence of the disease. While we recognize the potential challenge in drawing definitive conclusions from a limited sample, the observed high statistical significance in numerous parameters suggests the robustness of our findings. Nonetheless, future studies with a larger cohort of patients are warranted to enhance the generalizability of our results. Secondly, some patients were taking medication for their disease. However, none were taking medication chronically, and as mentioned above, the patients were free of any exacerbation at least three months before sampling, indicating that the influence of treatments on oxidative stress parameters can be considered negligible, although it cannot be completely ruled out. Thirdly, the absence of genetic analysis on disease-related mutations constitutes a significant limitation. This omission precludes our exploration of potential associations between specific mutations and oxidative stress levels. Incorporating genetic analyses in future investigations could provide valuable insights into the intricate relationship between genetic factors and oxidative stress in the PCD context, which could lead to the development of personalized therapeutic strategies and an improved long-term outcome for the affected individuals.

## 5. Conclusions

This study explores the intricate relationship between oxidative stress and PCD pathophysiology. Our results indicate a REDOX imbalance in PCD patients toward reductive stress that could be involved in the pathophysiology of the disease. The investigation of nasal epithelial ciliary cells reveals significant alterations in oxidative profiles, including decreased levels of nitric oxide and peroxynitrite, a paradoxical scenario in mitochondrial dynamics, and no significant differences in oxidized proteins or lipid ratios compared to healthy controls.

These findings contribute to a better understanding of PCD’s molecular mechanisms and highlight the need for further research to explore the interplay between symptoms and oxidative parameters in this clinical context. Further exploration into the consequences of this redox imbalance may offer valuable insights into the development and progression of PCD, ultimately contributing to identifying novel therapeutic approaches for managing this respiratory disorder.

## Figures and Tables

**Table 1:** Demographics and clinical–pathological characteristics in PCD patients and control individuals.

	Controls (n = 35)	Patients (n = 35)	*p*-Value
**Age (years ± SD)**	26.73 ± 9.51	27.09 ± 20.12	0.226
**Male/female (n/%)**	17 (49%)/18 (51%)	20 (57%)/15 (43%)	0.632
**BMI (kg m^−2^)**	20.00 ± 2.00	22.53 ± 5.00	0.520
***Situs inversus* (n/%)**	-	9 (26)	-
**Chronic cough (n/%)**	-	31 (89)	-
**Bronchiectasis (n/%)**	-	15 (43)	-
**Atelectasis (n/%)**	-	11 (31)	-
**Asthma (n/%)**	-	10 (29)	-
**Otitis (n/%)**	-	21 (60)	-
**Rhinosinusitis (n/%)**	-	18 (51)	-
**Pneumonia (n/%)**	-	20 (57)	-
**nNO (ppb)**	-	94.50 ± 64.50	-

Age is presented as mean and standard deviation. Sex is expressed as absolute values and as percentages of men and women. A comparison of proportions was performed using the chi-square test. Student’s *t*-test was used for mean comparison. A *p*-value < 0.05 was considered statistically significant. nNO: nasal nitric oxide; ppb: parts per billion.

**Table 2:** Ciliary ultrastructure defects in PCD patients.

Ciliary Ultrastructure Defects (n = 22)	Number of Patients	Percentage
Defects in both external and internal dynein arms	6	27
Defects only in internal dynein arms	7	32
Microtubule disorganization	2	9
Transposition	1	5
Partial dynein deficiency	3	14
Normal	3	14

**Table 3:** Ciliary beat pattern in PCD patients.

Ciliary Beat Pattern (n = 35)	Number of Patients	Percentage
Immobile cilia	9	26
Vibrational movement	11	31
Reduced amplitude and lack of coordination	9	26
Normal pattern but reduced frequency	6	17

**Table 4:** Biomarkers of oxidative stress in PCD patients and control individuals.

	Control (n = 35)	Patients (n = 35)	*p*-Value
**Apoptosis**	20.59 ± 18.97	10.01 ± 7.80	**0.004**
**ROS/RNS and antioxidant markers**			
GSH (FU)	1438.00 ± 507.40	1463.00 ± 653.70	0.867
O_2_^−^ (FU)	469.70 ± 406.70	273.00 ± 172.90	**0.018**
ONOO^−^ (FU)	29.04 ± 16.27	18.64 ± 14.24	**0.007**
NO (FU)	132.70 ± 165.60	53.66 ± 33.79	**0.007**
iCa^2+^	116.80 ± 53.55	111.20 ± 60.27	0.680
Plasma membrane potential	756.70 ± 767.00	748.10 ± 534.30	0.957
Intracellular peroxides	484.90 ± 409.20	823.20 ± 841.30	0.206
**Mitochondrial oxidative parameters**			
H_2_O_2_ (FU)	490.50 ± 237.40	270.10 ± 133.10	**<0.0001**
O_2_^−^ (FU)	155.10 ± 108.60	74.56 ± 63.04	**0.0004**
Mitochondrial mass (FU)	1723.00 ± 947.50	2314.00 ± 877.70	**0.009**
ΔΨm (FU)	590.50 ± 376.80	735.30 ± 536.60	0.200
**Markers of oxidative damage**			
Oxidized protein (FU)	1694.00 ± 1244.00	2147.80 ± 1298.00	0.1371
Oxidized/reduced lipid ratio (FU)	189.30 ± 163.70	178.90 ± 155.00	0.7973

Data are presented as mean and standard deviation. Bold indicates statistical significance (*p* < 0.05). GSH: reduced glutathione; GSSG: oxidized glutathione; O_2_^−^: superoxide anion; ONOO^−^: peroxynitrites; NO: nitric oxide; iCa^2+^: intracellular calcium; H_2_O_2_: hydrogen peroxide; ΔΨm: mitochondrial membrane potential; FU: fluorescence units.

## Data Availability

The data presented in this study are available on request from the corresponding author. The data are not publicly available due to privacy/ethical restrictions.

## Supplementary Materials

The following supporting information can be downloaded at https://www.mdpi.com/article/10.3390/antiox13020190/s1, Table S1: Oxidative stress parameters in PCD patients according to ciliary motility; Table S2: Oxidative stress parameters in PCD patients according to recurrent otitis; Table S3: Oxidative stress parameters in PCD patients according to recurrent pneumonia; Table S4: Oxidative stress parameters in PCD patients according to atelectasis; Table S5: Oxidative stress parameters in PCD patients according to bronchiectasis; Table S6: Oxidative stress parameters in PCD patients according to situs inversus; Table S7: Oxidative stress parameters in PCD patients according to chronic rhinosinusitis.

## References

[1] Hannah W.B., Seifert B.A., Truty R., Zariwala M.A., Ameel K., Zhao Y., Nykamp K., Gaston B. (2022). The Global Prevalence and Ethnic Heterogeneity of Primary Ciliary Dyskinesia Gene Variants: A Genetic Database Analysis. Lancet Respir. Med..

[2] Horani A., Ferkol T.W. (2021). Understanding Primary Ciliary Dyskinesia and Other Ciliopathies. J. Pediatr..

[3] Blanter M., Cockx M., Wittebols L., Salama S.A., Bondt M.D., Berghmans N., Pörtner N., Vanbrabant L., Lorent N., Gouwy M. (2022). Sputum from Patients with Primary Ciliary Dyskinesia Contains High Numbers of Dysfunctional Neutrophils and Inhibits Efferocytosis. Respir. Res..

[4] Pizzino G., Irrera N., Cucinotta M., Pallio G., Mannino F., Arcoraci V., Squadrito F., Altavilla D., Bitto A. (2017). Oxidative Stress: Harms and Benefits for Human Health. Oxidative Med. Cell. Longev..

[5] Boukhenouna S., Wilson M.A., Bahmed K., Kosmider B. (2018). Reactive Oxygen Species in Chronic Obstructive Pulmonary Disease. Oxidative Med. Cell. Longev..

[6] Dickerhof N., Pearson J.F., Hoskin T.S., Berry L.J., Turner R., Sly P.D., Kettle A.J., Arest C.F. (2017). Oxidative Stress in Early Cystic Fibrosis Lung Disease Is Exacerbated by Airway Glutathione Deficiency. Free Radic. Biol. Med..

[7] Escribano A., Pastor S., Reula A., Castillo S., Vicente S., Sanz F., Casas F., Torres M., Fernández-Fabrellas E., Codoñer-Franch P. (2016). Accelerated Telomere Attrition in Children and Teenagers with A1-Antitrypsin Deficiency. Eur. Respir. J..

[8] Escribano A., Amor M., Pastor S., Castillo S., Sanz F., Codoñer-Franch P., Dasí F. (2015). Decreased Glutathione and Low Catalase Activity Contribute to Oxidative Stress in Children with α-1 Antitrypsin Deficiency. Thorax.

[9] Magallón M., Castillo-Corullón S., Bañuls L., Pellicer D., Romero T., Martínez-Ferraro C., Navarro-García M.M., Herrejón A., González C., Dasí F. (2023). Hypoxia Enhances Oxidative Stress in Neutrophils from ZZ Alpha-1 Antitrypsin Deficiency Patients. Antioxidants.

[10] Zihlif N., Paraskakis E., Tripoli C., Lex C., Bush A. (2006). Markers of Airway Inflammation in Primary Ciliary Dyskinesia Studied Using Exhaled Breath Condensate. Pediatr. Pulmonol..

[11] Bodini A., Pecoraro L., Tenero L., Pezzella V., Pradal U., Piacentini G. (2021). Isoprostane-8 in the Exhaled Breath Condensate: Could It Represent a Noninvasive Strategic Tool for Primary Ciliary Dyskinesia Diagnosis and Management?. Lung India Off. Organ Indian Chest Soc..

[12] Lucas J.S., Barbato A., Collins S.A., Goutaki M., Behan L., Caudri D., Dell S., Eber E., Escudier E., Hirst R.A. (2017). European Respiratory Society Guidelines for the Diagnosis of Primary Ciliary Dyskinesia. Eur. Respir. J..

[13] Dalrymple R.A., Kenia P. (2019). European Respiratory Society Guidelines for the Diagnosis of Primary Ciliary Dyskinesia: A Guideline Review. Arch. Dis. Child.—Educ. Pr. Ed..

[14] Baz-Redón N., Rovira-Amigo S., Paramonov I., Castillo-Corullón S., Cols-Roig M., Antolín M., García-Arumí E., Torrent-Vernetta A., Messa I.d.M., Gartner S. (2021). Implementation of a Gene Panel for Genetic Diagnosis of Primary Ciliary Dyskinesia. Arch. Bronconeumol. (Engl. Ed.).

[15] Rubbo B., Shoemark A., Jackson C.L., Hirst R., Thompson J., Hayes J., Frost E., Copeland F., Hogg C., O’Callaghan C. (2019). Accuracy of High-Speed Video Analysis to Diagnose Primary Ciliary Dyskinesia. Chest.

[16] Reula A., Pellicer D., Castillo S., Magallón M., Armengot M., Herrera G., O’Connor J.-E., Bañuls L., Navarro-García M.M., Escribano A. (2021). New Laboratory Protocol to Determine the Oxidative Stress Profile of Human Nasal Epithelial Cells Using Flow Cytometry. J. Clin. Med..

[17] Noone P.G., Leigh M.W., Sannuti A., Minnix S.L., Carson J.L., Hazucha M., Zariwala M.A., Knowles M.R. (2004). Primary Ciliary Dyskinesia: Diagnostic and Phenotypic Features. Am. J. Respir. Crit. Care Med..

[18] Biswas S.K. (2016). Does the Interdependence between Oxidative Stress and Inflammation Explain the Antioxidant Paradox?. Oxidative Med. Cell. Longev..

[19] Paternò S., Pisani L., Zanconato S., Ferraro V.A., Carraro S. (2023). Role of Nasal Nitric Oxide in Primary Ciliary Dyskinesia and Other Respiratory Conditions in Children. Int. J. Mol. Sci..

[20] ben Anes A., Fetoui H., Bchir S., ben Nasr H., Chahdoura H., Chabchoub E., Yacoub S., Garrouch A., Benzarti M., Tabka Z. (2014). Increased Oxidative Stress and Altered Levels of Nitric Oxide and Peroxynitrite in Tunisian Patients with Chronic Obstructive Pulmonary Disease: Correlation with Disease Severity and Airflow Obstruction. Biol. Trace Elem. Res..

[21] Kanazawa H., Shiraishi S., Hirata K., Yoshikawa J. (2003). Imbalance between Levels of Nitrogen Oxides and Peroxynitrite Inhibitory Activity in Chronic Obstructive Pulmonary Disease. Thorax.

[22] Kanazawa H., Shiraishi S., Hirata K., Yoshikawa J. (2002). Decreased Peroxynitrite Inhibitory Activity in Induced Sputum in Patients with Bronchial Asthma. Thorax.

[23] Burkhalter M.D., Sridhar A., Sampaio P., Jacinto R., Burczyk M.S., Donow C., Angenendt M., Hempel M., Walther P., Competence Network for Congenital Heart Defects Investigators (2019). Imbalanced Mitochondrial Function Provokes Heterotaxy via Aberrant Ciliogenesis. J. Clin. Investig..

[24] Ignatenko O., Malinen S., Rybas S., Vihinen H., Nikkanen J., Kononov A., Jokitalo E.S., Ince-Dunn G., Suomalainen A. (2022). Mitochondrial Dysfunction Compromises Ciliary Homeostasis in Astrocytes. J. Cell Biol..

