# Supplementary material for: Redox Imbalance in Nasal Epithelial Cells of Primary Ciliary Dyskinesia Patients
Source: Antioxidants (Basel). 2024 Feb 2;13(2):190. doi: 10.3390/antiox13020190 (PMC10885940; doi:10.3390/antiox13020190)
Supplement: Supplementary file 1 [file antioxidants-13-00190-s001.zip › antioxidants-2785927-supplementary.pdf]

Supplementary Table S1. Oxidative stress parameters in PCD patients according to ciliary motility

Ciliary motility					
**Parameter**	**No motility (n=9)**	**Vibratile pattern (n=11)**	**Reduced motility (n=9)**	**Reduced frequency (n=6)**	**p-value**
**Apoptosis (%)**	10.83 ± 9.27	10.80 ± 7.91	5.92 ± 5.99	10.79 ± 8.69	0.531
**O_2^- (FU)**	237.20 ± 95.17	299.72 ± 199.40	188.40 ± 121.35	254.58 ± 232.87	0.764
**$ONNO^-$ (FU)**	22.05 ± 21.83	20.21 ± 12.44	10.58 ± 6.85	22.58 ± 9.19	0.194
**NO (FU)**	40.35 ± 23.68	64.83 ± 38.39	52.46 ± 2.12	51.10 ± 48.01	0.427
**mt H_2O_2 (FU)**	238.75 ± 92.82	276.18 ± 96.19	231.57 ± 77.20	280.60 ± 193.70	0.773
**mt O_2^- (FU)**	57.45 ± 36.73	91.92 ± 72.76	61.34 ± 33.06	59.63 ± 83.49	0.355
**mt mass (FU)**	2441.38 ± 1025.34	2142.00 ± 612.3	2403 ± 774.83	1986 ± 1314.24	0.762

Supplementary Table S2. Oxidative stress parameters in PCD patients according to recurrent otitis

Recurrent otitis			
**Parameter**	**No (n=14)**	**Yes (n=21)**	**p-value**
**Apoptosis (%)**	12.81 ± 9.18	9.35 ± 7.37	0.301
**O_2^- (FU)**	214.43 ± 118.34	281.63 ± 188.65	0.367
**$ONNO^-$ (FU)**	24.38 ± 14.68	17.79 ± 14.77	0.295
**NO (FU)**	46.63 ± 29.73	56.99 ± 34.61	0.462
**mt H_2O_2 (FU)**	270.38 ± 109.00	254.19 ± 107.68	0.721
**mt O_2^- (FU)**	60.73 ± 33.96	76.87 ± 68.04	0.530
**mt mass (FU)**	1987.75 ± 1104.32	2309.38 ± 799.46	0.378

Supplementary Table S3. Oxidative stress parameters in PCD patients according to recurrent pneumonia

Recurrent pneumonia			
**Parameter**	**No (n=15)**	**Yes (n=20)**	**p-value**
**Apoptosis (%)**	8.70 ± 8.70	10.12 ± 7.53	0.628
**O_2^- (FU)**	235.64 ± 98.86	264.77 ± 186.61	0.686
**$ONNO^-$ (FU)**	21.93 ± 18.75	15.75 ± 11.52	0.438
**NO (FU)**	45.62 ± 26.07	57.84 ± 36.48	0.336
**mt H_2O_2 (FU)**	235.82 ± 98.86	268.90 ± 114.06	0.426
**mt O_2^- (FU)**	62.28 ± 69.89	71.80 ± 54.06	0.911
**mt mass (FU)**	2220 ± 913.34	2271 ± 864.24	0.877

Supplementary Table S4. Oxidative stress parameters in PCD patients according to atelectasis

	Atelectasis		
**Parameter**	**No (n=24)**	**Yes (n=11)**	**p-value**
**Apoptosis (%)**	12.42 ± 8.84	6.52 ± 4.75	0.052
**O_2^- (FU)**	288.74 ± 189.75	198.29 ± 118.48	0.191
**$ONNO^-$ (FU)**	20.00 ± 14.74	20.35 ± 14.71	0.953
**NO (FU)**	50.28 ± 32.28	59.70 ± 36.15	0.472
**mt H_2O_2 (FU)**	274.17 ± 118.44	233.09 ± 81.97	0.322
**mt O_2^- (FU)**	78.27 ± 72.04	62.85 ± 35.06	0.515
**mt mass (FU)**	2090.72 ± 994.09	2483.36 ± 675.54	0.259

Supplementary Table S5. Oxidative stress parameters in PCD patients according to bronchiectasis

	Bronchiectasis		
**Parameter**	**No (n=20)**	**Yes (n=15)**	**p-value**
**Apoptosis (%)**	11.23 ± 7.21	9.66 ± 8.93	0.616
**O_2^- (FU)**	248.91 ± 129.24	272.16 ± 203.64	0.417
**$ONNO^-$ (FU)**	20.45 ± 11.89	21.17 ± 16.58	0.898
**NO (FU)**	58.88 ± 38.58	51.23 ± 29.78	0.560
**mt H_2O_2 (FU)**	251.69 ± 97.43	264.80 ± 120.14	0.756
**mt O_2^- (FU)**	84.18 ± 66.16	65.02 ± 56.86	0.418
**mt mass (FU)**	2091.54 ± 748.06	2273.73 ± 948.26	0.589

Supplementary Table S6. Oxidative stress parameters in PCD patients according to chronic rhinosinusitis

	Chronic rhinosinusitis		
**Parameter**	**No (n=17)**	**Yes (n=18)**	**p-value**
**Apoptosis (%)**	12.02 ± 8.99	8.70 ± 7.05	0.266
**O_2^- (FU)**	283.24 ± 154.63	228.85 ± 182.85	0.425
**$ONNO^-$ (FU)**	27.30 ± 17.01	14.08 ± 9.68	**0.013**
**NO (FU)**	61.48 ± 36.72	46.85 ± 30.84	0.248
**mt H_2O_2 (FU)**	279.17 ± 93.70	236.33 ± 116.37	0.296
**mt O_2^- (FU)**	89.90 ± 67.30	58.09 ± 52.75	0.158
**mt mass (FU)**	2134.17 ± 856.91	2312.72 ± 911.09	0.598

Supplementary Table S7. Oxidative stress parameters in PCD patients according to *situs inversus*

	Situs inversus		
**Parameter**	**No (n=26)**	**Yes (n=9)**	**p-value**
**Apoptosis (%)**	11.26 ± 4.87	5.31 ± 4.87	0.053
**O_2^- (FU)**	262.64 ± 18.88	237.59 ± 99.04	0.740
**$ONNO^-$ (FU)**	19.63 ± 13.88	17.68 ± 17.21	0.760
**NO (FU)**	55.58 ± 35.97	47.54 ± 24.76	0.564
**mt H_2O_2 (FU)**	260.83 ± 114.01	247.88 ± 96.97	0.784
**mt O_2^- (FU)**	73.86 ± 66.02	62.45 ± 33.25	0.645
**mt mass (FU)**	2213.87 ± 916.93	2366.63 ± 748.02	0.675
